# Dietary supplementation of coated sodium butyrate improves growth performance of laying ducks by regulating intestinal health and immunological performance

**DOI:** 10.3389/fimmu.2023.1142915

**Published:** 2023-03-09

**Authors:** Tao Zeng, Hanxue Sun, Manman Huang, Rongbing Guo, Tiantian Gu, Yongqing Cao, Chengfeng Li, Yong Tian, Li Chen, Guoqin Li, Lizhi Lu

**Affiliations:** ^1^ State Key Laboratory for Managing Biotic and Chemical Threats to the Quality and Safety of Agro-Products, Institute of Animal Husbandry and Veterinary Science, Zhejiang Academy of Agricultural Sciences, Hangzhou, China; ^2^ College of Animal Science, Zhejiang A&F University, Hangzhou, China; ^3^ Hubei Shendan Health Food Co., Ltd., Xiaogan, China

**Keywords:** coated sodium butyrate (CSB), laying duck, growth performance, intestinal health, immunological performance

## Abstract

**Introduction:**

This study was conducted to assess the effects of dietary supplementation of coated sodium butyrate (CSB) on the growth performance, serum antioxidant, immune performance, and intestinal microbiota of laying ducks.

**Methods:**

A total of 120 48-week-old laying ducks were randomly divided into 2 treatment groups: the control group (group C fed a basal diet) and the CSB-treated group (group CSB fed the basal diet + 250 g/t of CSB). Each treatment consisted of 6 replicates, with 10 ducks per replicate, and the trial was conducted for 60 days.

**Results:**

Compared with the group C, the group CSB showed a significant increase in the laying rate (p<0.05) of the 53-56 week-old ducks. Additionally, the serum total antioxidant capacity, superoxide dismutase activity and immunoglobulin G level were significantly higher (p<0.05), while the serum malondialdehyde content and tumor necrosis factor (TNF)-a level were significantly lower (p<0.05) in the serum of the group CSB compared to the group C. Moreover, the expression of IL-1b and TNF-a in the spleen of the group CSB was significantly lower (p<0.05) compared to that of the group C. In addition, compared with the group C, the expression of Occludin in the ileum and the villus height in the jejunum were significantly higher in the group CSB (p<0.05). Furthermore, Chao1, Shannon, and Pielou-e indices were higher in the group CSB compared to the group C (p<0.05). The abundance of Bacteroidetes in the group CSB was lower than that in the group C (p<0.05), while the abundances of Firmicutes and Actinobacteria were higher in the group CSB compared to the group C (p<0.05).

**Conclusions:**

Our results suggest that the dietary supplementation of CSB can alleviate egg-laying stress in laying ducks by enhancing immunity and maintaining the intestinal health of the ducks.

## Introduction

For a long time, animal feeds were supplemented with antibiotics to promote growth and disease resistance. However, this practice has since been banned, due to the discovery of the complications associated with the unregulated use of antibiotics (1), thereby necessitating the discovery of green and safe feed additives for poultry to improve their antioxidant capacity and reduce disease incidence (2). Oxidative Stress (OS) is a state of imbalance between oxidative and antioxidant effects in the body, and it is clear from previous studies that OS can also lead to follicular atresia and ovarian senescence, reducing animal reproductive performance (3, 4). It has been shown that OS can affect nutrient metabolism through deleterious effects on intestinal function, gut microbial flora and altered dynamic homeostasis in poultry (5, 6). At the peak of egg laying, ducks lay eggs almost every day, and in order to meet the nutritional needs of the output ducks themselves, the intake increases accordingly, resulting in OS, which creates egg laying stress thus affecting egg production (7).

Short Chain Fatty Acids (SCFAs) are organic fatty acids with a carbon chain length of 1-6, and sodium butyrate (SB), an SCFA produced during fermentation, is an important nutrient for the intestinal cells, providing for >70% of the energy requirements (8, 9). SB has been widely used as a feed additive for pigs (10) and recently for poultry (11). However, SB is less palatable as a feed additive due to its unpleasant odor and volatility. Coating SB in palm oil (coated sodium butyrate, CSB) improves its palatability, allowing its use as a feed additive (12). SB-supplementation is thought to improve the development (13, 14) and morphological structure of the intestinal mucosa and regulate the growth of commensal intestinal flora. In addition, butyric acid (BA) can mediate the immune response, inhibit the growth of harmful bacteria, induce the proliferation and differentiation of intestinal epithelium, and protect epithelial cells (15, 16). Moreover, CSB can regulate the development of many immune cells, such as macrophages (17), and the expression of inflammatory cytokines, including interleukin (IL)-6, IL-8, interferon (IFN)-γ, transforming growth factor (TGF)-β, and IL-1β (18). Several studies have reported the promoting effects of SB in improving immunity and small intestinal structure, to regulate intestinal flora in poultry (19–21).

However, studies on the potential effects of CSB on laying animals, especially laying ducks, are limited. Considering the previous reports on the beneficial effects of CSB on poultry, we suspect that adding CSB in diet can increase immunity and antioxidant capacity of laying ducks and reduce laying stress. To test this hypothesis, we studied the effects of CSB on growth performance, immune performance and intestinal health of laying ducks.

## Materials and methods

### Animals and experimental design

The CSB (30% SB covered with palm oil) was purchased from Hangzhou King Techina Feed Co., Ltd., (Hangzhou, China). Shendan No. 2 laying ducks (obtained by a ternary cross between Shaoxing, Jinyun, and Youxian duck varieties), used for commercial duck egg production, were provided by Hubei Shendan Health Food Co., Ltd. (Hubei, China).

A total of 120 48-week-old Shendan No. 2 laying ducks were randomly divided into 2 treatment groups: the control group (group C), which was fed a basal diet and the CSB-treated group (group CSB), which was fed the basal diet supplemented with 250 g/t of CSB. Each treatment consisted of 6 replicates with 10 ducks per replicate. The feeding trials were conducted in a closed duck house at the welfare duck farm of Hubei Shendan Health Food Co., Ltd. The ducks were housed in four layers of cages (50 ×50 ×60 cm) at 2 ducks/cage, and the replicates of the same treatment were evenly distributed in the duck house. During the experimental period, the duck house was maintained at 20-25 °C, 65-75% relative humidity, and 16:8 h light/dark cycle (4:00-20:00 h). The basic feed of the experimental ducks combined with the needs of ducks during the egg-laying period met the nutritional requirements of the Chinese egg and duck standard (GB/T 41189-2021), and the basic composition of the feed was shown in **Table 1**. The ducks were fed twice a day (8:00 and 14:00) by hand, from 48 to 56 weeks of age.

**Table 1:** Composition and nutrient levels of basal diets (air-dry basis) %.

Ingredients	Content	Nutrient levels	Content
Corn	42.0	ME/(MJ/kg)^2^	11.72
Soybean meal	29.0	CP	18.80
Rice bran	4.0	Lys	1.06
Wheat bran	2.1	Met	0.48
Soybean oil	2.5	Met+Cys	0.82
Flour	10.0	Trp	0.24
Limestone	5.8	Ca	3.36
Fine gravel	2.0	TP	0.59
CaHPO_4_	1.1	AP	0.31
Chaff	0.2		
NaCl	0.3		
Premix^1^	1.0		
Total	100		

^1^The premix provided the following per kg of diet: Vitamin (V)_A_ 12500 IU, V_D3_ 3500 IU, V_E_ 20 IU, V_K3_ 2.65 mg, V_B1_ 2.00 mg, V_B2_ 6.00 mg, V_B6_ 3.00 mg, V_B12_ 0.025 mg, Biotin 0.0325 mg, Folic acid 12.00 mg, Pantothenic acid 50.00 mg, Niacin 50.00 mg, Cu 6 mg, Fe 80 mg, Zn 40 mg, Mn 100 mg, Se 0.15 mg, and I 0.35 mg.

^2^ME was a calculated value, while the others were measured values.

### Bird slaughter and sample collection

The number of eggs laid and the weight of the feed, in each replicate, were recorded daily, and the weight of the leftover feed in each replicate was recorded weekly, to evaluate the growth performance of laying ducks. For sampling, 6 56-week-old ducks were chosen per treatment (one duck from each replicate), after 12 h of fasting. All the experiments and methods were designed to minimize animal suffering. Blood collected from the wing vein was dispensed into 5 mL procoagulation tubes for 3-4 h and centrifuged at 3,000 rpm for 5 min. The serum was collected and transferred to 1.5 mL EP tubes and stored at -20 °C. After slaughter, spleen, ileum tissues were collected immediately, placed into sterilized freeze tubes, and stored at -80 °C after flash-freezing in liquid nitrogen. Thereafter, 12 ducks were randomly selected per treatment (2 ducks per replicate, with 6 previously sampled ducks), and duodenum, jejunum, and ileum segments were collected in EP tubes and fixed using 4% paraformaldehyde, and cecal contents were collected immediately, the treatment is the same as the above organization.

### Growth performance

As mentioned earlier, the number of eggs laid and the feed weight, per each replicate, were recorded daily, and the leftover feed per each replicate was recorded weekly, to evaluate the average daily feed intake (ADFI), laying rate, and feed-to-egg ratio (F/E), as follows:


ADFI (g) = feed intake (g)/days

Laying rate (%) = number of eggs/days/100%

Feed-to-eggratio(F/E)=feedintake(g)/eggsweight(g)

### Serum antioxidant status and cytokine analysis

Total antioxidant capacity kit, No. HY-60021 was used to detect total antioxidant capacity (T-AOC), activities of superoxide dismutase kit, No. HY-M0001 was used to detect the activities of serum superoxide dismutase (SOD), catalase kit, No. HY-M0018 was used to detect the catalase (CAT), content of malondialdehyde kit, No. HY-M0003 was used to detect the malondialdehyde (MDA) were determined according to the manufacturer’s instructions accompanying the assay kit (Beijing Huaying Biotechnology Research Institute, Beijing, China).

Immunoglobulin G kit, No. bs-0293G was used to detect the immunoglobulin G (IgG), immunoglobulin A kit, No. bs-0360G was used to detect the immunoglobulin A (IgA), immunoglobulin M kit, No. bs-0314P was used to detect the immunoglobulin M (IgM), interferon gamma kit, No. bs-0481P was used to detect the interferon gamma (IFN-γ), interleukin-6 kit, No. bs-0379P was used to detect the interleukin-6 (IL-6), Interleukin-1β kit, No. bs-0812P was used to detect the Interleukin-1β (IL-1β), tumor necrosis factor-α, No. bs-0078P was used to detect the tumor necrosis factor (TNF)-α in serum segments were determined by absorbance changes at 450 nm with ELISA kits (Beijing Huaying Biotechnology Research Institute, Beijing, China) according to the manufacturer’s protocol. Concentrations of immunoglobulin and cytokine were calculated with the standard curve.

### Immunity and intestinal barrier-related gene expression

#### RNA Extraction

RNA was extracted using the Total RNA kit I (22) R6834-01 (Omega, Norcross, Georgia, USA). Approximately 20 mg of spleen tissue (or ileum tissue), 500 mL of lysis buffer, and 2 sterilized steel beads were added to a 1.5 mL EP tube and homogenized using an automatic sample grinder (Shanghai Jingxin Technology Co., Ltd., Shanghai, China). Thereafter, the supernatant was transferred to a centrifuge column for 3 min, and the filtrate was transferred to a 1.5 mL EP tube with 2x (v/v) of 70% ethanol. After shaking with a vortex (Vortex Genie2, Scientific Industries Inc, NY, USA) the liquid was transferred to a HiBind RNA microcolumn and centrifuged for 3 min and the supernatant was discarded. After adding Buffer I, the samples were centrifuged for 2 min and the supernatant was discarded. Thereafter, this step was repeated twice with Buffer II. Lastly, the eluent was added to the centrifuge for 2 mins to obtain the extracted RNA. The quality of the extracted RNA was measured using a Nanodrop (NanoDrop One, Thermo, USA) by measuring the OD 260/280 values, and the RNA was stored at -80°C for subsequent analyses.

#### Reverse transcription of total RNA

Genomic DNA was isolated and added to RNase-free EP tubes. Thereafter, the sample was subjected to reverse transcription using the HiScript^®^ II Q RT SuperMix for qPCR (+gDNA wiper) kit (R223-01, Nanjing Novozymes Biotechnology Co., Ltd., Nanjing, China).

#### Fluorescent quantitative polymerase chain reaction

Fluorescent quantitative PCR was conducted using the ChamQ Universal SYBR qPCR Master Mix (Q711-02/03, Nanjing Novozymes Biotechnology Co., Nanjing, China). β-actin was used as an internal control, and the relative gene expression was calculated by the 2^-△△Ct^ method. The primer sequences were obtained from the previous literature or primer design software (**Table 2**), and they were synthesized by Shanghai Jereh Bioengineering Co. (Shanghai, China).

**Table 2:** Primer Sequence.

Gene	Primer Sequence (5’-3’)	Product size/bp
*IL-1β*	F:GCTTCATCTTCTACCGCCTGGAC	159
	R:TTAGCTTGTAGGTGGCGATGTTGAC’	
*IL-6*	F:TCTGGCAACGACGATAAGG	154
	R:TGAAGTAAAGTCTCGGAGGATG	
*IFN-γ*	F:ATACCCTTTCCAATGACT	130
	R:GTCTCCACCAGTTTCTGT	
*TNF-α*	F:AAATCTGCTGCTGGTCTT	235
	R:CCATCATCGTCCTCACTA	
*ZO-1*	F: GGGGAAGACAACTGATGC	159
	R: TTGTGATGTGCTGGGAGA	
*Claudin-1*	F: TGATGGTGGCTGCGATAC	218
	R: AACAGGCGTGAAAGGGTC	
*Occludin*	F: GCAGGATGTGGCAGAGGAATA	119
	R: TGCGCTTGATGTGGAAGAGTT	
*β-actin*	F:CCCCATTGAACACGGTATTGTC	151
	R:GGCTACATACATGGCTGGGG	

#### Small intestinal morphometric traits

Each intestinal segment was fixed in 4% paraformaldehyde for more than 24 h (EG1150h, LEIC, Germany) and smoothed using a scalpel, in a fume hood. The tissues were then subjected to dehydration and embedded in wax using an embedding machine (rotating microbodies, RM2225, LEIC, Germany). Thereafter, the sections were dewaxed and stained with hematoxylin and eosin (H&E) (23). The villus height (VH) and crypt depth (CD) were observed under a light microscope (S4E, LEIC, Germany) and analyzed using an Image analyzer (Image-Proplus 5.0).

#### Cecum microbiome

After the above-mentioned cecal content DNA was extracted, 16S rRNA sequencing technology was used to analyze the diversity composition spectrum of the cecal content microbial community in Shanghai Personal Biotechnology Co., Ltd. (Shanghai, China). The detailed steps and analysis process are described in the previous research article (24).

### Statistical analysis

Statistical analysis was conducted using the SPSS software package (SPSS version 22.0; IBM Corp, Armonk, NY, United States). The Shapiro-Wilk test was used for the phenotypic analyses, and the Student’s t-test was used to analyze the differences after compound normal distribution conditions.

## Results

### Determination of growth performance

**Figure 1:**
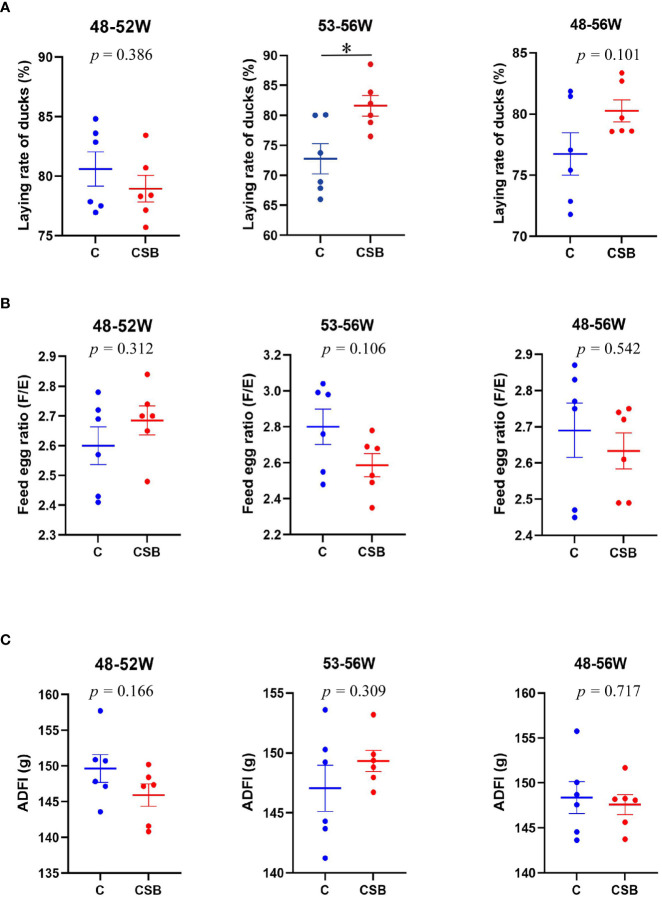
Effects of dietary supplementation of coated sodium butyrate (CSB) on the growth performance of laying ducks. **(A)** Laying rate of different times laying ducks. **(B)** Feed-to-egg ratio (F/E) of different times laying ducks. **(C)** Average daily feed intake (ADFI) of different times laying ducks. Values are presented as means ± SEM (n = 6). *p<0.05. group C, Control group, which was fed a basal diet; CSB group, CSB-treated group, which was fed the basal diet supplemented with 250 g/t of CSB.

The effects of dietary CSB supplementation on the growth performance, including laying rate, F/E ratio, and ADFI of the 48-56 week-old laying ducks are shown in **Figures 1A-C**. The results showed that dietary CSB supplementation improves ADFI and F/E of the ducks, although there is no significant difference between the groups C and CSB (*p >*0.05; **Figures 1B, C** **)**. In contrast, the laying rate of the group CSB was significantly higher than that of the group C (*p<*0.05; **Figure 1A**).

### Determination of serum antioxidant index

**Figure 2:**
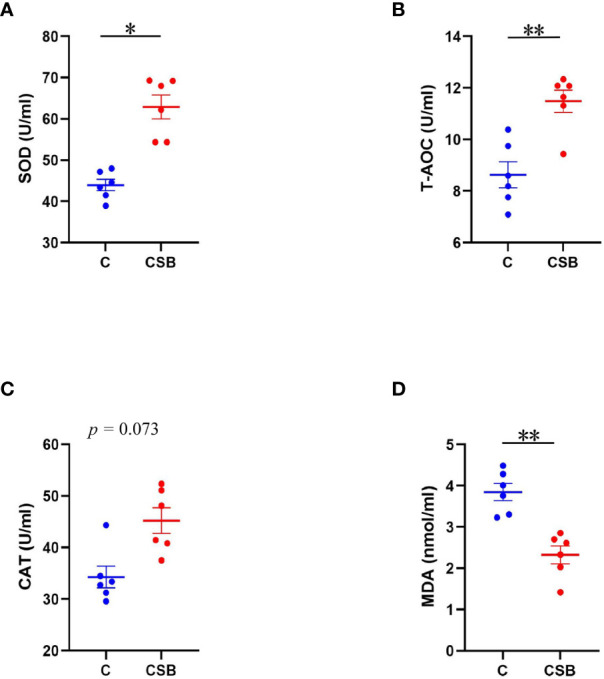
Effects of dietary supplementation of coated sodium butyrate (CSB) on the serum antioxidant status of laying ducks. **(A)** Superoxide dismutase (SOD). **(B)** Total antioxidant capacity (T-AOC). **(C)** Catalase (CAT), and **(D)** Malondialdehyde (MDA). Values are presented as means ± SEM (n = 6). *p<0.05 and **p<0.01. group C, Control group, which was fed a basal diet; CSB group, CSB-treated group, which was fed the basal diet supplemented with 250 g/t of CSB.

The effect of dietary CSB supplementation on the serum antioxidant indices of laying ducks is shown in **Figures 2A-D**. The SOD and T-AOC activity were significantly higher, while the MDA content was significantly lower in the serum of the group CSB compared to that of the group C (*p<*0.01; **Figures 2A, B, D** **)**. The results showed that dietary CSB supplementation improves CAT of the ducks, although there is no significant difference between the groups C and CSB (*p >*0.05; **Figure 2C**).

### Serum Ig and inflammatory cytokine analysis

**Figure 3:**
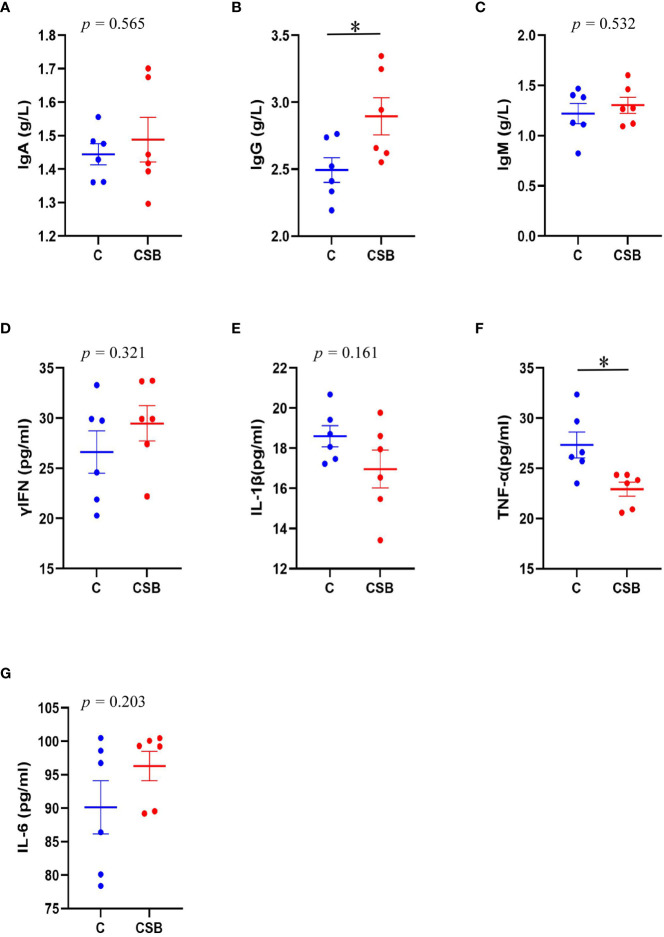
Effects of dietary supplementation of coated sodium butyrate (CSB) on the serum immunoglobulin **(Ig)** and inflammatory cytokine levels of laying ducks. **(A)** IgA. **(B)** IgG. **(C)** IgM. **(D)** Interferon (IFN)-γ. **(E)** Interleukin (IL)-B. **(F)** Tumor necrosis factor (TNF)-α. **(G)** Interleukin (IL)-6. Values are presented as means ± SEM (n = 6). *p<0.05. group C, Control group, which was fed a basal diet; CSB group, CSB-treated group, which was fed the basal diet supplemented with 250 g/t of CSB.

The effect of dietary CSB supplementation on the serum Igs and inflammatory cytokines of laying ducks is shown in **Figures 3A-G**. The results showed that dietary CSB supplementation increases the serum IgA and IgM levels, although there was no significant difference between the groups C and CSB (*p >*0.05; **Figures 3A, C** **)**. In contrast, compared to the group C, the group CSB showed a significant increase in serum IgG levels (*p<*0.05; **Figure 3B**). The serum TNF-α content was significantly lower in the group CSB compared with that in the group C (*p<*0.05; **Figure 3F**).

### Analysis of spleen immune-related gene and ileal tight junction protein gene expression

**Figure 4:**
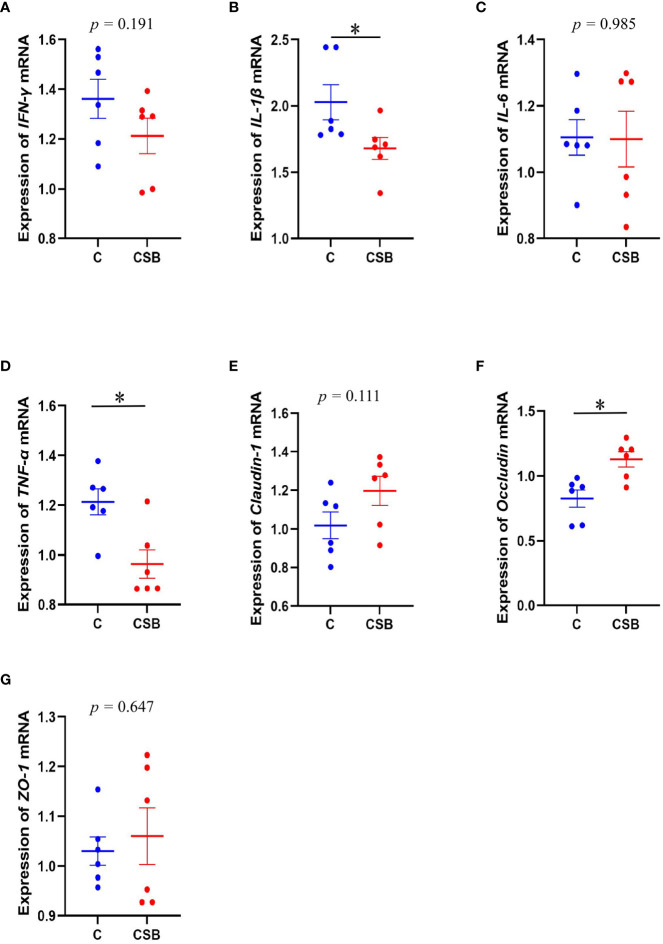
Effects of dietary supplementation of coated sodium butyrate (CSB) on the expression of immune-related genes in the spleen and the ileal tight junction protein gene of laying ducks. **(A)** *Interferon-γ* (*IFN-γ*). **(B)** *Interleukin-1β* (*IL-1β*). **(C)** *Interleukin-6* (*IL-6*). **(D)** Tumor necrosis factor alpha (*TNF-α*). **(E)** *Claudin-1.* **(F)** *Occludin.* **(G)** *ZO-1.* Values are presented as means ± SEM (n = 6). *p<0.05. group C, Control group, which was fed a basal diet; CSB group, CSB-treated group, which was fed the basal diet supplemented with 250 g/t of CSB.

The effect of dietary CSB supplementation on the expression of immune-related genes in the spleen and ileal TJ protein of laying ducks is shown in **Figures 4A-G**. The expression of *IL-1β* and *TNF-α* in the spleen of the group CSB was significantly lower compared to that in the group C (*p<*0.05; **Figures 4B, D** **)**. In contrast, there was no significant difference between the expression of *IFN-γ* and *IL-6* in the spleen of the groups C and CSB (*p >*0.05; **Figures 4A, C** **)**. The expression of *Occludin* in the ileum of the group CSB was significantly higher than that in the group C (*p<*0.05; **Figure 4F**). Furthermore, *ZO-1* and *Claudin-1* expression were increased in the ileum of the group CSB, although the difference between the two groups was not significant (*p >*0.05; **Figures 4E, G** **)**.

### Small intestinal morphology examination

**Figure 5:**
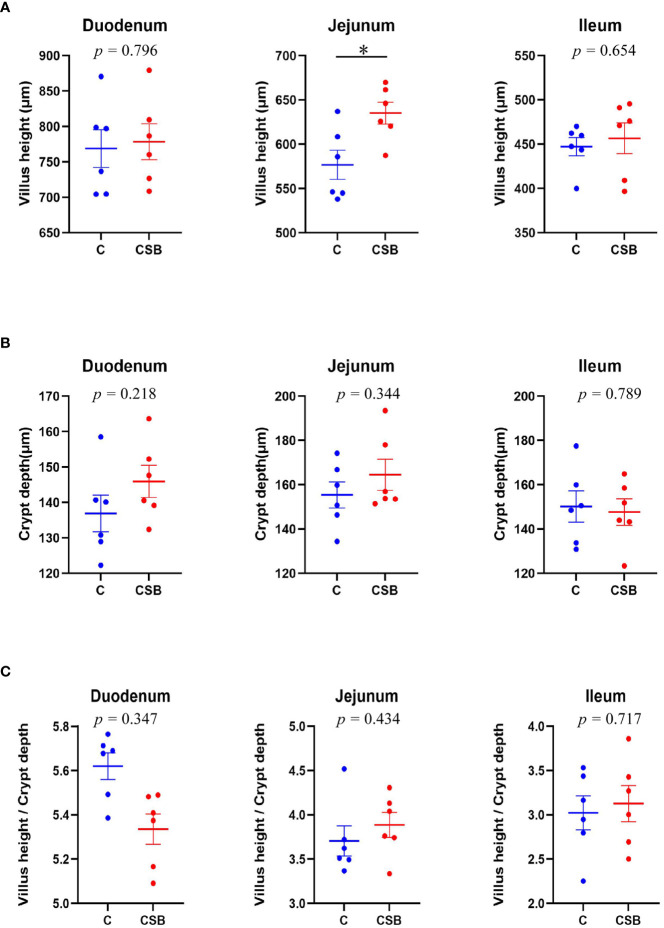
Effects of dietary supplementation of coated sodium butyrate (CSB) on the morphometric characteristics of the duodenum, jejunum, and ileum of laying ducks. **(A)** Villus height (VH) of small intestines. **(B)** Crypt depth (CD) of small intestines. **(C)** VH/CD ratio of the small intestines. Values are presented as means ± SEM (n = 12). *p<0.05. group C, Control group, which was fed a basal diet; CSB group, CSB-treated group, which was fed the basal diet supplemented with 250 g/t of CSB.

**Figure 6:**
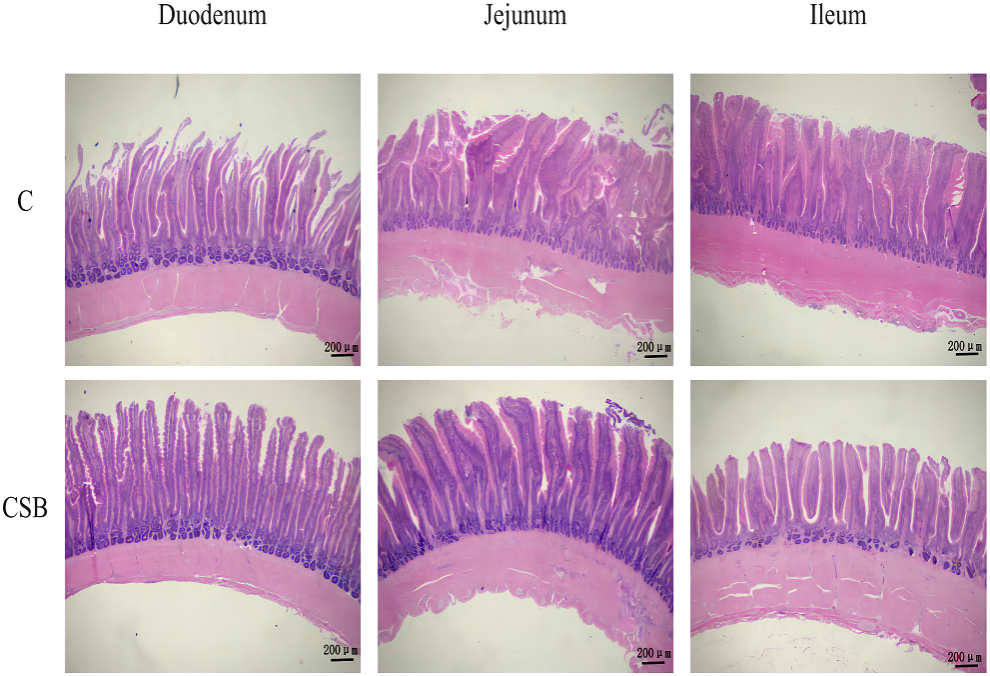
Effects of dietary supplementation of coated sodium butyrate (CSB) on the intestinal (duodenal, jejunal, and ileal) morphology of laying ducks. Intestinal sections were stained with hematoxylin and eosin (H&E). group C, Control group, which was fed a basal diet; CSB group, CSB-treated group, which was fed the basal diet supplemented with 250 g/t of CSB.

The jejunal VH in the group CSB was significantly higher (*p<*0.05) than that in the group C (**Figure 5A**). However, there was so significant difference between the villus morphology of the duodenum and ileum of the groups C and CSB (*p >*0.05; **Figures 5B, C** **)**. H&E stained images of the small intestines (duodenum, jejunum, and ileum) of the groups C and CSB are shown in **Figure 6**.

### Cecal microbiome analysis

**Figure 7:**
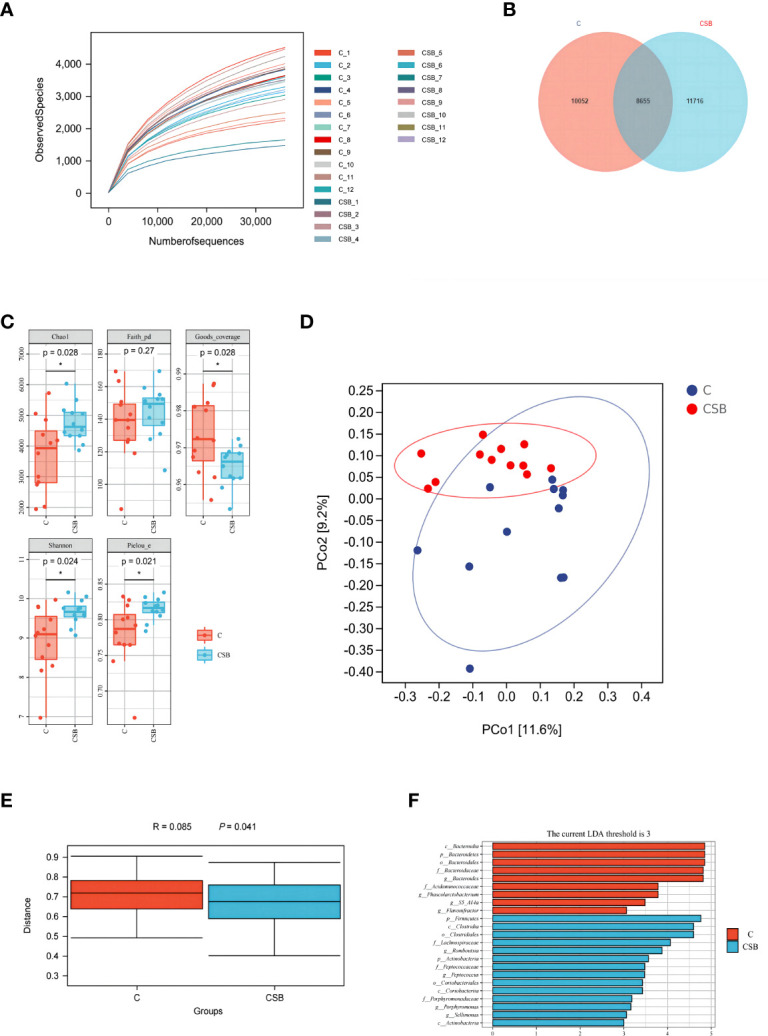
Effects of dietary supplementation of coated sodium butyrate (CSB) on cecum microbiota in laying ducks. **(A)** Rarefaction curves. **(B)** Venn diagram of the amplicon sequence variants (ASVs). **(C)** Chao1, Faith’s phylogenetic diversity (PD), Good’s coverage, Shannon, and Pielou’s evenness (Pielou-e) α-diversity indices. **(D)** principal coordinate analysis (PCoA) of taxonomical classifications of the cecal bacterial communities. **(E)** weighted_unifrac distance. **(F)** linear discriminant analysis effect size (LEfSe) analysis (LDA threshold = 3). Values are presented as means ± SEM (n = 12). *p<0.05. group C, Control group, which was fed a basal diet; CSB group, CSB-treated group, which was fed the basal diet supplemented with 250 g/t of CSB.

A total of 1,229,199 bases were obtained from 24 samples, which were quality filtered and chimera removed, to obtain a total of 30,423 *amplicon sequence variants (ASVs)*. Rarefaction analyses were performed to gauge adequate sequencing depth per sample (**Figure 7A**). The Venn diagram in **Figure 7B** shows that the ducks in the *groups C and CSB* had 10,051 and 11,716 unique ASVs (**Figure 7B**), respectively. The α-diversity assessed using Chao1, Faith*’*s PD, Good*’*s coverage, Shannon, and Pielou-e indices is shown in **Figure 7C**. The Chao1, Shannon, and Pielou-e indices were higher in the *group CSB* than in the *group C* (p<0.05), while the Good*’*s coverage index was higher in the *group C* compared to the *group CSB* (p<0.05). These results suggest that compared to the *group C*, cecal microflora richness and diversity were higher in the *group CSB*.

The PCoA plot was used to visualize the microbial trends and outliers to determine the difference in the gut microbial composition of the groups C and CSB (**Figure 7D**). Analysis of similarities (ANOSIM) indicated a clear difference between the two groups (**Figure 7E**). Analysis of the differentially abundant taxa between the two groups revealed that *c:Bacteroidia*, *p_Bacteroidetes*, *o_Bacteroidales*, *f_Bacteroidaceae*, *and g_Bacteroides* were enriched in the group C (*p<*0.05), while *p_Firmicutes*, *c_Clostridia*, *o_Clostridiales*, and *f_Lachnospiraceae* were enriched in the CBS group (*p<*0.05; **Figure 7F**).

**Figure 8:**
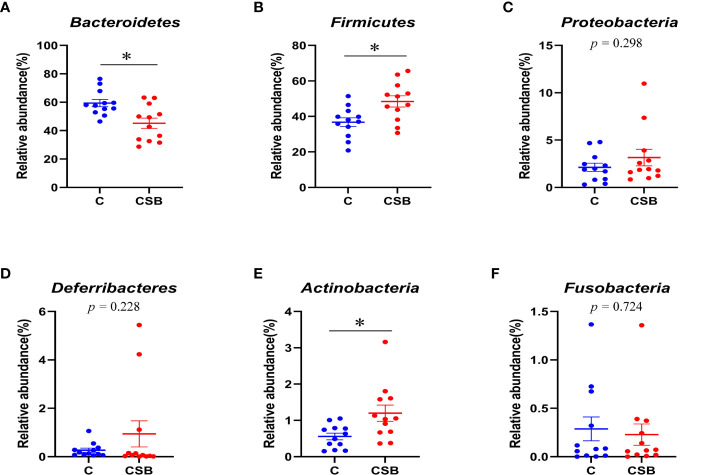
Effects of dietary supplementation of coated sodium butyrate (CSB) on the relative abundance of cecal microbiota of laying ducks at the genus level. **(A)** *Bacteroides.* **(B)** *Faecalibacterium.* **(C)** *Megamonas.* **(D)** *Subdoligranulum.* **(E)** *Desulfovibrio.* **(F)** *Prevotellaceae_Ga6A1_group.* Values are presented as means ± SEM (n = 12). *p<0.05. group C, Control group, which was fed a basal diet; CSB group, CSB-treated group, which was fed the basal diet supplemented with 250 g/t of CSB.

**Figure 9:**
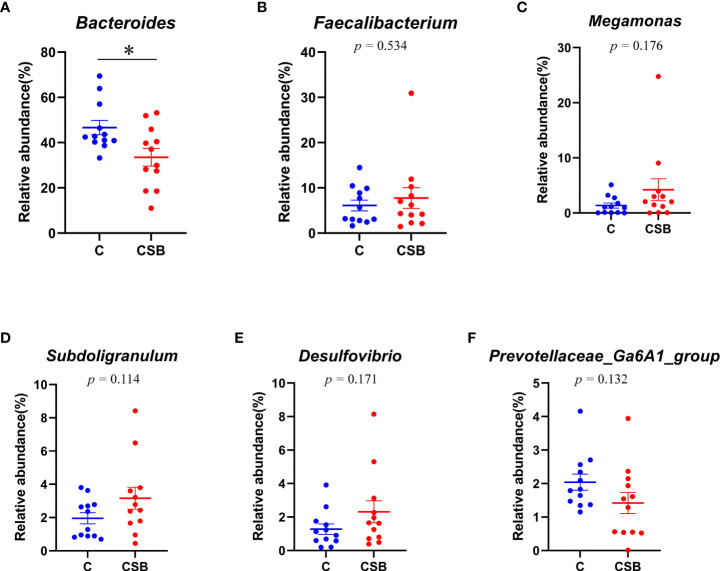
Effects of dietary supplementation of coated sodium butyrate (CSB) on the relative abundance of the cecal microbiota of laying ducks at the phylum level. **(A)** *Bacteroidetes.* **(B)** *Firmicutes.* **(C)** *Proteobacteria.* **(D)** *Deferribacteres.* **(E)** *Actinobacteria.* **(F)** *Fusobacteria.* Values are presented as means ± SEM (n = 12). *p<0.05. group C, Control group, which was fed a basal diet; CSB group, CSB-treated group, which was fed the basal diet supplemented with 250 g/t of CSB.

The cecal microflora community structure of the groups C and CSB was nearly identical at the phylum level (**Figures 8A-F**). In the group C, *Bacteroidetes* were the most abundant phyla (59.41%), followed by *Firmicutes* (36.77%), *Proteobacteria* (2.12%), and *Actinobacteria* (0.57%). In contrast, in the group CSB, *Firmicutes* were the most abundant phyla (48.44%), followed by *Bacteroidetes* (45.19%), *Proteobacteria* (3.16%), and *Actinobacteria* (1.2%). Furthermore, based on the variance analysis, we found that the abundance of *Bacteroidetes* in the group CSB was lower than that in the group C (*p<*0.05; **Figure 9A**), while the abundances of *Firmicutes* and *Actinobacteria* were higher in the group CSB than in the group C (*p<*0.05; **Figures 8B, E** **)**. In contrast, at the genus level, both groups C and CSB showed similar trends (**Figures 9A-F**), with *Bacteroides* being the most abundant (46.65% and 33.50%), followed by *Faecalibacterium* (6.10% and 7.74%), *Megamonas* (1.36% and 4.22%), and *Subdoligranulum* (1.95% and 3.17%). However, the abundance of *Bacteroides* in the group CSB was lower than that in the group C (*p<*0.05; **Figure 9A**).

## Discussion

Cultural backgrounds and dietary habits have led to an increase in the demand for meat and duck eggs in Asia, and duck egg, along with hen, has become an important component of the food industry owing to its excellent nutritional and functional properties (25). Asia is the world’s largest producer of duck meat and eggs (26), with China being the primary producer (27). However, with the transformation of the breeding models and increasing market demand, healthy breeding has become the top priority in the animal husbandry sector. Some studies have shown that SB is beneficial to the reproductive performance of the hens (28, 29). Furthermore, CSB was found to increase the laying rate and the daily egg weight, while decreasing the F/E ratio of yellow-feathered breeder hens (30). This is consistent with the results of the present study, in which the laying rate of the CSB-supplemented ducks increased significantly. According to Ghosh and Cox (31), SB induced follicle-stimulating hormone secretion, stimulated follicle growth and development, and increased the laying rate. Moreover, CSB supplementation improved the ADFI and F/E ratio of laying ducks, although there was no significant variation between the groups C and CSB, suggesting that the effect of butyrate was too low in the pre-test period to make a significant difference.

OS often occurs in animals during production, and the main reason for its production is the production and accumulation of reactive oxygen species (ROS) (32).In order to fight this oxidative stress, the body uses antioxidant enzymes to limit the accumulation of ROS and reduce the stress response (33). T-AOC is a comprehensive indicator of the antioxidant function of the body, and the serum MDA level reflects the degree of FR-mediated lipid peroxidation (34). Numerous studies have shown that butyrate has a powerful antioxidant effect both inside (35–38). Some studies have shown that dietary CSB supplementation can increase SOD and T-AOC activities and reduce MDA levels in laying hens (19). In our study, the serum T-AOC and SOD activity were significantly higher, while the MDA content was significantly lower in the group CSB compared to the control group, indicating that dietary CSB supplementation can improve the serum antioxidant capacity of laying duck.

Immunoglobulins produced by B lymphocytes bind specifically to the corresponding antigen (39). Immunoglobulins have the largest proportion of IgG in serum, probably because of their specific immune activity (40). Dietary SB supplementation increases the serum IgG content in meat-type chickens (41) and weaned piglets (42). However, only limited studies have been published on the effects of SB on the humoral and cellular immune status (43, 44), and further research is required on this aspect. In our study, the serum IgG content of the group CSB was significantly higher than that of the control group, which improved the humoral immune capacity of the group CSB. The pro-inflammatory cytokines, including IL-1β, IL-6, IFN-γ, and TNF-α, are closely associated with the immune system and are influenced by the highly-automated process (HAP) axis activity (45). BA has been shown to play an important role in maintaining the integrity of the intestinal mucosa and exert potent anti-inflammatory effects in broilers (46). Previous studies have also reported that butyrate modulates the immune system by stimulating the release of inflammatory cytokines (47). Zou et al. (48), found that TNF-α and IL-1β were significantly decreased in chickens supplemented with 300 mg/kg SB, which is consistent with the results of this study, in which dietary CSB supplementation significantly decreased serum TNF-α levels in laying ducks.

Furthermore, previous studies have demonstrated that supplementation of BA or SB reduces the expression of pro-inflammatory cytokines and affects immune response by inhibiting the activation of nuclear factor-κB (NF-κB) and decreasing the release of IL-1β, IL-6, and TNF-α (49). Moreover, dietary supplementation of CSB can significantly suppress the intestinal gene expression levels of pro-inflammatory cytokines, TNF-α and IL-6, in laying hens (19). This is consistent with the results of our study, in which compared with the control group, the expression of *IL-1β* and *TNF-α* was significantly lower in the spleens of the group CSB. These results suggest that CSB can affect cellular inflammatory factors and thus the immune capacity of the organism. Homeostasis in the intestines can be maintained by a physical barrier consisting of epithelial cells and intercellular TJs (50). TJs are the most important intercellular junctions and consist of cytoplasmic protein *ZO* family and transmembrane proteins, including *Occludin* and *Claudin* (22). Previous studies demonstrated that SB increased TJ expression, reduced gut permeability, and enhanced the intestinal physical barrier (20, 21). This is consistent with the results of the present study, in which dietary CSB supplementation significantly increased the expression of *Occludin* in the ileum of laying ducks. Intestinal permeability is closely associated with cytokines (51), and TNF-α and IL-6 are enriched in the inflamed gut and contribute to gut damage (52). The results of our study suggest that CSB can inhibit cellular inflammatory factors, thereby enhancing the physical barrier of the intestines and maintaining intestinal integrity.

In addition to the barrier, intestinal tract histology also plays an important role in laying ducks. The absorption capacity of the small intestine is measured by VH, CD, and, VH/CD ratio (53). The longer villi correspond to a shallower crypt and greater VH/CD ratio, which increases the absorptive capacity of the small intestines (54). Previous studies found that SB supplementation could improve feed utilization by increasing the length of the small intestinal villi (41, 55), which is consistent with the results of this study, in which CSB supplementation significantly increased the VH in the jejunum of the group CSB compared to the control group. Furthermore, BA expands the absorption area of the small intestines, thereby increasing the VH (56).

The cecum contains majority of the intestinal flora and plays a key role in the digestion and absorption process of poultry (57). Some studies have shown that changes in the intestinal flora can affect intestinal health (58), and the cecal microbiota composition significantly impacts the growth and health of poultry (59, 60). A study found that the addition of *Clostridium butyricum* to the feeds of early Muscovy ducks improved the cecal microflora richness (61). In this study, we found that CSB supplementation increased the Chao1, Shannon, and Pielou-e indices in laying ducks, suggesting higher cecal microflora richness and diversity in the group CSB compared to the control group. Higher microbiota diversity indicates stronger intestinal health, as the rich flora can resist the invasion of pathogenic bacteria (62). Whether or not to add CSB to the ration the dominant flora of the cecum of ducks are *Bacteroidetes* and *Firmicutes*, this is consistent with the results of previous studies (59, 63). *C. butyricum* can increase the abundance of *Firmicutes* in the cecum of yellow-feathered breeder hens (30). In our study, we found that the abundance of *Bacteroidetes* was lower in the group CSB than in the control group, while the abundances of *Firmicutes* and *Actinobacteria* were higher in the group CSB compared to the control group. *Firmicutes* are involved in the energy absorption activities of the intestines (64) and include a variety of bacteria that can decompose cellulose in the intestinal tract (65), *Bacteroidetes* can degrade polysaccharides in the large intestine to produce butyrate (66). Due to CSB supplementation, *Bacteroidetes* were not required for butyrate production to maintain the normal activity of the organism, which explains the significantly lower abundance of *Bacteroidetes* in the group CSB compared to the control group, which is consistent with the results of our previous studies (67, 68). Furthermore, in our previous study, we found that CSB increased the abundance of *Actinobacteria* in the ileum of suckling pigeons (12), which is consistent with the results of this pilot study. *Actinobacteria* have great economic importance owing to their secondary metabolites, which have antibiotic properties (69). CSB increases egg production by affecting the intestinal flora of the ducks, thereby increasing their immune and antioxidant capacity.

## Conclusions

Dietary supplementation of CSB enhanced the immune function and intestinal barrier of laying ducks by increasing the antioxidant capacity of the body, increasing the secretion of humoral immune factors, upregulating the expression of TJ-related genes, and improving intestinal morphology. Moreover, CSB supplementation improved the abundance of cecal microflora in laying ducks, reduced laying stress, and increased egg production rate, which indicates the positive effect of CSB supplementation on laying ducks.

## Data availability statement

The datasets presented in this study can be found in online repositories. The names of the repository/repositories and accession number(s) can be found below: https://ngdc.cncb.ac.cn, PRJCA014034.

## Ethics statement

The animal care and use protocol was approved by the Ethics Committee for the Welfare of Animals of Zhejiang Academy of Agricultural Sciences (NO. 2022ZAASLA59).

## Author contributions

LL and TZ designed the experiments. HS and MH performed the experiments and carried out the data summarizing. RG and TG were mainly responsible for experimental animal feeding and result analysis. TZ, HS, YC, CL, YT, CL and GL wrote and revised the main manuscript. All authors contributed to the article and approved the submitted version.

## References

[1] Luo D Li J Xing T Zhang L Gao F. Combined effects of xylo-oligosaccharides and coated sodium butyrate on growth performance, immune function, and intestinal physical barrier function of broilers. Anim Sci J (2021) 92:e13545. doi: 10.1111/asj.13545 33793035

[2] Casewell M Friis C Marco E McMullin P Phillips I. The European ban on growth-promoting antibiotics and emerging consequences for human and animal health. J Antimicrob Chemother (2003) 52:159–61 doi: 10.1093/jac/dkg313 12837737

[3] Wang J Jia R Celi P Zhuo Y Ding X Zeng Q. Resveratrol alleviating the ovarian function under oxidative stress by alternating microbiota related tryptophan-kynurenine pathway. Front Immunol (2022) 13:911381. doi: 10.3389/fimmu.2022.911381 35911670 PMC9327787

[4] Shen M Lin F Zhang J Tang Y Chen WK Liu H. Involvement of the up-regulated FoxO1 expression in follicular granulosa cell apoptosis induced by oxidative stress. J Biol Chem (2012) 287:25727–40. doi: 10.1074/jbc.M112.349902 PMC3406661 22669940

[5] Wang J Jia R Gong H Celi P Zhuo Y Ding X. The effect of oxidative stress on the chicken ovary: Involvement of microbiota and melatonin interventions. Antioxidants (Basel) (2021) 10:1422. doi: 10.3390/antiox10091422 PMC8472688 34573054

[6] Li Y Wang P Yin J Jin S Su W Tian J. Effects of ornithine alpha-ketoglutarate on growth performance and gut microbiota in a chronic oxidative stress pig model induced by d-galactose. Food Funct (2020) 11:472–82. doi: 10.1039/C9FO02043H 31833510

[7] Jing B Xiao H Yin H Wei Y Wu H Zhang D. Feed supplemented with aronia melanocarpa (AM) relieves the oxidative stress caused by ovulation in peak laying hens and increases the content of yolk precursors. Anim (Basel) (2022) 12:3574. doi: 10.3390/ani12243574 PMC9774901 36552494

[8] Bedford A Gong J. Implications of butyrate and its derivatives for gut health and animal production. Anim Nutr (2018) 4:151–9. doi: 10.1016/j.aninu.2017.08.010 PMC6104520 30140754

[9] Hamer HM Jonkers D Venema K Vanhoutvin S Troost FJ Brummer RJ. Review article: the role of butyrate on colonic function. Aliment Pharmacol Ther (2008) 27:104–19. doi: 10.1111/j.1365-2036.2007.03562.x 17973645

[10] Jang YD Lindemann MD Monegue HJ Monegue JS. The effect of coated sodium butyrate supplementation in sow and nursery diets on lactation performance and nursery pig growth performance. Livest Sci (2017) 195:13–20. doi: 10.1016/j.livsci.2016.11.005

[11] Wu W Xiao Z An W Dong Y Zhang B. Dietary sodium butyrate improves intestinal development and function by modulating the microbial community in broilers. PloS One (2018) 13:e197762. doi: 10.1371/journal.pone.0197762 PMC5967726 29795613

[12] Sun H Liu Y Zeng T Li G Tao Z Zhou X. Effects of coated sodium butyrate and polysaccharides from cordyceps cicadae on intestinal tissue morphology and ileal microbiome of squabs. Front Vet Sci (2022) 9:813800. doi: 10.3389/fvets.2022.813800 35310408 PMC8931417

[13] Smulikowska S Czerwiński J Mieczkowska A Jankowiak J. The effect of fat-coated organic acid salts and a feed enzyme on growth performance, nutrient utilization, microflora activity, and morphology of the small intestine in broiler chickens. J Anim Feed Sci (2009) 18:478–89. doi: 10.22358/jafs/66422/2009

[14] Wu Y Zhou Y Lu C Ahmad H Zhang H He J. Influence of butyrate loaded clinoptilolite dietary supplementation on growth performance, development of intestine and antioxidant capacity in broiler chickens. PloS One (2016) 11:e154410. doi: 10.1371/journal.pone.0154410 PMC4841535 27104860

[15] Elnesr SS Alagawany M Elwan H Fathi MA Farag MR. Effect of sodium butyrate on intestinal health of poultry – a review. Sciendo (2020) 20:29–41 doi: 10.2478/aoas-2019-0077

[16] Liu J Zhu H Li B Lee C Alganabi M Zheng S. Beneficial effects of butyrate in intestinal injury. J Pediatr Surg (2020) 55:1088–93. doi: 10.1016/j.jpedsurg.2020.02.036 32234318

[17] Sunkara LT Achanta M Schreiber NB Bommineni YR Dai G Jiang W. Butyrate enhances disease resistance of chickens by inducing antimicrobial host defense peptide gene expression. PloS One (2011) 6:e27225. doi: 10.1371/journal.pone.0027225 22073293 PMC3208584

[18] Xu J Chen X Yu S Su Y Zhu W. Effects of early intervention with sodium butyrate on gut microbiota and the expression of inflammatory cytokines in neonatal piglets. PloS One (2016) 11:e162461. doi: 10.1371/journal.pone.0162461 PMC5017769 27611998

[19] Miao S Hong Z Jian H Xu Q Liu Y Wang X. Alterations in intestinal antioxidant and immune function and cecal microbiota of laying hens fed on coated sodium butyrate supplemented diets. Anim (Basel) (2022) 12:545. doi: 10.3390/ani12050545 PMC8908843 35268114

[20] Wu X Wang L Xie Q Tan P. Effects of dietary sodium butyrate on growth, diet conversion, body chemical compositions and distal intestinal health in yellow drum (Nibea albiflora, Richardson). Aquac Res (2020) 51:69–79. doi: 10.1111/are.14348

[21] Wang HB Wang PY Wang X Wan YL Liu YC. Butyrate enhances intestinal epithelial barrier function *via* up-regulation of tight junction protein claudin-1 transcription. Dig Dis Sci (2012) 57:3126–35. doi: 10.1007/s10620-012-2259-4 22684624

[22] Turner JR. Intestinal mucosal barrier function in health and disease. Nat Rev Immunol (2009) 9:799–809. doi: 10.1038/nri2653 19855405

[23] Murugesan GR Syed B Haldar S Pender C. Corrigendum II: Phytogenic feed additives as an alternative to antibiotic growth promoters in broiler chickens. Front Vet Sci (2016) 3:28. doi: 10.3389/fvets.2016.00028 27092306 PMC4819148

[24] Sun H Du X Zeng T Ruan S Li G Tao Z. Effects of compound probiotics on cecal microbiome and metabolome of shaoxing duck. Front Microbiol (2021) 12:813598. doi: 10.3389/fmicb.2021.813598 35087506 PMC8787150

[25] Quan TH Benjakul S. Duck egg albumen: physicochemical and functional properties as affected by storage and processing. J Food Sci Technol (2019) 56:1104–15. doi: 10.1007/s13197-019-03669-x PMC6423336 30956290

[26] Huang JF Pingel H Guy G Ukaszewicz E Wang SD. A century of progress in waterfowl production, and a history of the WPSA waterfowl working group. World’s Poultry Sci J (2012) 68:551–63. doi: 10.1017/S0043933912000645

[27] Zeng T Chen L Du X Lai SJ Huang SP Liu YL. Association analysis between feed efficiency studies and expression of hypothalamic neuropeptide genes in laying ducks. Anim Genet (2016) 47:606–9. doi: 10.1111/age.12457 27329478

[28] Jahanian R Golshadi M. Effect of dietary supplementation of butyric acid glycerides on performance, immunological responses, ileal microflora, and nutrient digestibility in laying hens fed different basal diets. Livest Sci (2015) 178:228–36. doi: 10.1016/j.livsci.2015.05.038

[29] Zhan HQ Dong XY Li LL Zheng YX Gong YJ Zou XT. Effects of dietary supplementation with clostridium butyricum on laying performance, egg quality, serum parameters, and cecal microflora of laying hens in the late phase of production. Poult Sci (2019) 98:896–903. doi: 10.3382/ps/pey436 30285187

[30] Wang Y Wang Y Lin X Gou Z Fan Q Jiang S. Effects of clostridium butyricum, sodium butyrate, and butyric acid glycerides on the reproductive performance, egg quality, intestinal health, and offspring performance of yellow-feathered breeder hens. Front Microbiol (2021) 12:657542. doi: 10.3389/fmicb.2021.657542 34603221 PMC8481923

[31] Ghosh NK Cox RP. Induction of human follicle-stimulating hormone in HeLa cells by sodium butyrate. Nature (1977) 267:435–7. doi: 10.1038/267435a0 876359

[32] Ding X Cai C Jia R Bai S Zeng Q Mao X. Dietary resveratrol improved production performance, egg quality, and intestinal health of laying hens under oxidative stress. Poult Sci (2022) 101:101886. doi: 10.1016/j.psj.2022.101886 35526444 PMC9092510

[33] Li Y Wei L Cao J Qiu L Jiang X Li P. Oxidative stress, DNA damage and antioxidant enzyme activities in the pacific white shrimp (Litopenaeus vannamei) when exposed to hypoxia and reoxygenation. Chemosphere (2016) 144:234–40. doi: 10.1016/j.chemosphere.2015.08.051 26363325

[34] Orchel A Gruchlik A Weglarz L Dzierzewicz Z. Influence of sodium butyrate on antioxidative enzymes activity in caco-2 cell lines. Acta Pol Pharm (2006) 63:441–2. 17357609

[35] Russo I Luciani A De Cicco P Troncone E Ciacci C. Butyrate attenuates lipopolysaccharide-induced inflammation in intestinal cells and crohn’s mucosa through modulation of antioxidant defense machinery. PloS One (2012) 7:e32841. doi: 10.1371/journal.pone.0032841 22412931 10.1371/journal.pone.0032841 PMC3295784

[36] Ma X Fan PX Li LS Qiao SY Zhang GL Li DF. Butyrate promotes the recovering of intestinal wound healing through its positive effect on the tight junctions. J Anim Sci (2012) 90 Suppl 4:266–8. doi: 10.2527/jas.50965 23365351

[37] Lin Y Fang ZF Che LQ Xu SY Wu D Wu CM. Use of sodium butyrate as an alternative to dietary fiber: effects on the embryonic development and anti-oxidative capacity of rats. PloS One (2014) 9:e97838. doi: 10.1371/journal.pone.0097838 24852604 PMC4031178

[38] Jiang Y Zhang WH Gao F Zhou GH. Micro-encapsulated sodium butyrate attenuates oxidative stress induced by corticosterone exposure and modulates apoptosis in intestinal mucosa of broiler chickens. Anim Prod Sci (2014) 55:587. doi: 10.1071/AN13348

[39] Luo Q Cui H Peng X Fang J Zuo Z. Intestinal IgA+ cell numbers as well as IgA, IgG, and IgM contents correlate with mucosal humoral immunity of broilers during supplementation with high fluorine in the diets. Biol Trace Elem Res (2013) 154:62–72. doi: 10.1007/s12011-013-9713-9 23740525

[40] Jania B Andraszek K. Application of native agarose gel electrophoresis of serum proteins in veterinary diagnostics. J Vet Res (2016) 60:501–8. doi: 10.1515/jvetres-2016-0074

[41] Makled MN Abouelezz K Gad-Elkareem A Sayed AM. Comparative influence of dietary probiotic, yoghurt, and sodium butyrate on growth performance, intestinal microbiota, blood hematology, and immune response of meat-type chickens. Trop Anim Health Prod (2019) 51:2333–42. doi: 10.1007/s11250-019-01945-8 31168683

[42] Fang CL Sun H Wu J Niu HH Feng J. Effects of sodium butyrate on growth performance, haematological and immunological characteristics of weanling piglets. J Anim Physiol Anim Nutr (Berl) (2014) 98:680–5. doi: 10.1111/jpn.12122 24024579

[43] Ahsan U Cengiz Ö Raza I Kuter E Chacher MFA Iqbal Z. Sodium butyrate in chicken nutrition: the dynamics of performance, gut microbiota, gut morphology, and immunity. World’s Poultry Sci J (2016). 72:265–275 doi: 10.1017/S0043933916000210

[44] Sikandar A Zaneb H Younus M Masood S Aslam A Khattak F. Effect of sodium butyrate on performance, immune status, microarchitecture of small intestinal mucosa and lymphoid organs in broiler chickens. Asian-Australas J Anim Sci (2017) 30:690–9. doi: 10.5713/ajas.16.0824 PMC5411829 28111438

[45] Touchette KJ Carroll JA Allee GL Matteri RL Dyer CJ Beausang LA. Effect of spray-dried plasma and lipopolysaccharide exposure on weaned pigs: I. Effects Immune axis weaned pigs. J Anim Sci (2002) 80:494–501. doi: 10.2527/2002.802494x 11881933

[46] Zhou ZY Packialakshmi B Makkar SK Dridi S Rath NC. Effect of butyrate on immune response of a chicken macrophage cell line. Vet Immunol Immunopathol (2014) 162:24–32. doi: 10.1016/j.vetimm.2014.09.002 25278494

[47] Zhang H Du M Yang Q Zhu MJ. Butyrate suppresses murine mast cell proliferation and cytokine production through inhibiting histone deacetylase. J Nutr Biochem (2016) 27:299–306. doi: 10.1016/j.jnutbio.2015.09.020 26601598

[48] Zou X Ji J Qu H Wang J Shu DM Wang Y. Effects of sodium butyrate on intestinal health and gut microbiota composition during intestinal inflammation progression in broilers. Poult Sci (2019) 98:4449–56. doi: 10.3382/ps/pez279 31162611

[49] Song M Xia B Li J. Effects of topical treatment of sodium butyrate and 5-aminosalicylic acid on expression of trefoil factor 3, interleukin 1beta, and nuclear factor kappaB in trinitrobenzene sulphonic acid induced colitis in rats. Postgrad Med J (2006) 82:130–5. doi: 10.1136/pgmj.2005.037945 PMC2596699 16461476

[50] Vicente Y Da RC Yu J Hernandez-Peredo G Martinez L Perez-Mies B. Architecture and function of the gastroesophageal barrier in the piglet. Dig Dis Sci (2001) 46:1899–908. doi: 10.1023/A:1010631030320 11575442

[51] Andrews C McLean MH Durum SK. Cytokine tuning of intestinal epithelial function. Front Immunol (2018) 9:1270. doi: 10.3389/fimmu.2018.01270 29922293 PMC5996247

[52] Neurath MF. Cytokines in inflammatory bowel disease. Nat Rev Immunol (2014) 14:329–42. doi: 10.1038/nri3661 24751956

[53] Zhang C Chen KK Zhao XH Wang C Geng ZY. Effect of l-theanine on the growth performance, immune function, and jejunum morphology and antioxidant status of ducks. Animal (2019) 13:1145–53. doi: 10.1017/S1751731118002884 30376911

[54] Viveros A Chamorro S Pizarro M Arija I Centeno C Brenes A. Effects of dietary polyphenol-rich grape products on intestinal microflora and gut morphology in broiler chicks. Poult Sci (2011) 90:566–78. doi: 10.3382/ps.2010-00889 21325227

[55] Zhang WH Jiang Y Zhu QF Gao F Dai SF Chen J. Sodium butyrate maintains growth performance by regulating the immune response in broiler chickens. Br Poult Sci (2011) 52:292–301. doi: 10.1080/00071668.2011.578121 21732874

[56] Kotunia A Wolinski J Laubitz D Jurkowska M Rome V Guilloteau P. Effect of sodium butyrate on the small intestine development in neonatal piglets fed (correction of feed) by artificial sow. J Physiol Pharmacol (2004) 55 Suppl 2:59–68. 15608361

[57] Liu L Zhao X Wang Q Sun X Xia L Wang Q. Prosteatotic and protective components in a unique model of fatty liver: Gut microbiota and suppressed complement system. Sci Rep (2016) 6:31763. doi: 10.1038/srep31763 27550859 PMC4994046

[58] Wu Y Liu W Li Q Li Y Yan Y Huang F. Dietary chlorogenic acid regulates gut microbiota, serum-free amino acids and colonic serotonin levels in growing pigs. Int J Food Sci Nutr (2018) 69:566–73. doi: 10.1080/09637486.2017.1394449 29141471

[59] Wang JM Gan XM Pu FJ Wang WX Ma M Sun LL. Effect of fermentation bed on bacterial growth in the fermentation mattress material and cecum of ducks. Arch Microbiol (2021) 203:1489–97. doi: 10.1007/s00203-020-02145-x 33398398

[60] Lunedo R Furlan LR Fernandez-Alarcon MF Squassoni GH Campos D Perondi D. Intestinal microbiota of broilers submitted to feeding restriction and its relationship to hepatic metabolism and fat mass: Fast-growing strain. J Anim Physiol Anim Nutr (Berl) (2019) 103:1070–80. doi: 10.1111/jpn.13093 30934145

[61] Xiao X Fu Z Li N Yang H Wang W Lyu W. Modulation of the intestinal microbiota by the early intervention with clostridium butyricum in Muscovy ducks. Antibiotics (Basel) (2021) 10:836. doi: 10.3390/antibiotics10070826 PMC8300754 34356746

[62] Wen J Zhao W Li J Hu C Zou X Dong X. Dietary supplementation of chitosan oligosaccharide-clostridium butyricum synbiotic relieved early-weaned stress by improving intestinal health on pigeon squabs (Columba livia). Front Immunol (2022) 13:926162. doi: 10.3389/fimmu.2022.926162 35844624 PMC9284028

[63] Liu J Stewart SN Robinson K Yang Q Lyu W Whitmore MA. Linkage between the intestinal microbiota and residual feed intake in broiler chickens. J Anim Sci Biotechnol (2021) 12:22. doi: 10.1186/s40104-020-00542-2 33573700 PMC7879522

[64] Ley RE Turnbaugh PJ Klein S Gordon JI. Microbial ecology: human gut microbes associated with obesity. NATURE (2006) 444:1022–3. doi: 10.1038/4441022a 17183309

[65] Zhu Y Sun Y Wang C Li F. Impact of dietary fibre:starch ratio in shaping caecal archaea revealed in rabbits. J Anim Physiol Anim Nutr (Berl) (2017) 101:635–40. doi: 10.1111/jpn.12585 27561235

[66] Chen J Yu B Chen D Zheng P Luo Y Huang Z. Changes of porcine gut microbiota in response to dietary chlorogenic acid supplementation. Appl Microbiol Biot (2019) 103:8157–68. doi: 10.1007/s00253-019-10025-8 31401751

[67] Li C Chen J Zhao M Liu M Yue Z Liu L. Effect of sodium butyrate on slaughter performance, serum indexes and intestinal barrier of rabbits. J Anim Physiol Anim Nutr (Berl) (2022) 106:156–66. doi: 10.1111/jpn.13571 34096104

[68] Hongjin LI Sha W Yin B Lianjun FU Liu D Guojiang LI. Effects of oated sodium butyrate on intestinal health and growth performance of weaned piglets. J Domest Anim Ecol (2017) 38:30–4. doi: 10.3969/j.issn.1673-1182.2017.09.006

[69] Crippen TL Sheffield CL Singh B Byrd JA Beier RC Anderson RC. Poultry litter and the environment: Microbial profile of litter during successive flock rotations and after spreading on pastureland. Sci Total Environ (2021) 780:146413. doi: 10.1016/j.scitotenv.2021.146413 33774310

